# Predictive factors of incidental prostate cancer in patients undergoing surgery for presumed benign prostatic hyperplasia: an updated systematic review and meta-analysis

**DOI:** 10.3389/fonc.2025.1561675

**Published:** 2025-02-27

**Authors:** Yang Wang, Xiancheng Li, Hua Yang, Chaoshan Yin, Yameng Wu, Xiaoke Chen

**Affiliations:** ^1^ Department of Urology, Guangdong Provincial People’s Hospital, Zhuhai Hospital (Jinwan Central Hospital of Zhuhai), Zhuhai, China; ^2^ Department of Urology, The Second Affiliated Hospital of Dalian Medical University, Dalian, China; ^3^ Department of Urology, Renmin Hospital, Hubei University of Medicine, Shiyan, China; ^4^ Department of Urology, Fuyang Hospital of Anhui Medical University, Fuyang, China

**Keywords:** incidental prostate cancer, benign prostatic hyperplasia, meta-analysis, risk factors, surgery

## Abstract

**Purpose:**

We aimed to identify the clinical predictors of incidental prostate cancer (IPCa) after surgery for presumed benign prostatic hyperplasia (BPH).

**Methods:**

The literature was comprehensively searched using PubMed, Web of Science, Embase, and Cochrane databases in December 2024. We used pooled standardized mean difference (SMD) and odds ratio (OR) to describe the correlation between relevant risk factors and IPCa.

**Results:**

Twenty-one studies included 10,842 patients that were available for further analysis. After BPH surgery, 957 patients were histopathologically diagnosed with IPCa. The IPCa rate was 8.83%. Most importantly, our results identified that IPCa was significantly associated with age (pooled SMD = 0.36, *P* < 0.001), body mass index (BMI) (pooled SMD = 0.23, *P* < 0.001), preoperative prostate-specific antigen (pre-PSA) (pooled SMD = 0.43, *P* < 0.001), preoperative prostate-specific antigen density (pre-PSAD) (pooled SMD = 0.62, *P* = 0.028), resected prostate weight (pooled SMD = -0.22, *P* < 0.001), preoperative treatment with 5-alpha reductase inhibitors (5αRIs) (yes/no) (pooled OR = 0.60, *P* < 0.001), family history (yes/no) (pooled OR = 3.81, *P* = 0.029), digital rectal examination (DRE) findings (abnormal/normal) (pooled OR = 5.15, *P* < 0.001), and transrectal ultrasonography (TRUS) findings (abnormal/normal) (pooled OR = 2.92, *P* < 0.001). Additionally, sensitivity and subgroup analyses indicated that our findings were reliable and robust. However, we found no significant associations between IPCa and prostate volume, preoperative negative prostate biopsy, smoking history, history of hypertension, history of diabetes, history of dyslipidemia, and abnormal magnetic resonance imaging findings (all *P* > 0.05).

**Conclusions:**

Age, BMI, pre-PSA, pre-PSAD, resected prostate weight, preoperative treatment with 5αRIs, family history, abnormal DRE findings, and abnormal TRUS findings are independent factors predicting IPCa following BPH surgery. Before BPH surgery, factors such as age, BMI, pre-PSA, and pre-PSAD should be considered to assess the risk of IPCa. For high-risk patients, more detailed imaging and needle biopsy are recommended before surgery to avoid missed diagnosis. In the future, more large-scale and well-designed studies are needed to validate our results further.

**Systematic review registration:**

https://www.crd.york.ac.uk/prospero/, identifier CRD42025631346.

## Introduction

Prostate cancer (PCa) is the second most common cancer among men, accounting for 14.2% of all cancers (1). Incidental prostate cancer (IPCa) refers to the discovery of PCa during the histopathological analysis of resected prostate tissue that was initially assumed to be benign prostatic hyperplasia (BPH) (2). For men without clinical signs of PCa or with negative biopsy results, transurethral resection of the prostate (TURP) is the preferred treatment for lower urinary tract symptoms due to BPH when medication is ineffective. However, the final pathology examination may still reveal PCa as an incidental finding. With the introduction of prostate-specific antigen (PSA) screening, there has been a dramatic fall in IPCa incidence (3). For patients receiving surgical treatment for BPH without a previous PCa diagnosis, the incidence of IPCa after TURP decreased from 14.9% to 5.2% (4). Although most IPCa cases are clinically insignificant, slow-growing, and have a low risk of progression, some can be aggressive and clinically significant (5). Anract et al. (5) conducted a multicenter retrospective study involving 2,452 patients and found that 10.0% of patients were diagnosed with IPCa after BPH surgery, of which 20.2% were clinically significant; for patients with clinically insignificant IPCa, active surveillance was recommended by most international guidelines (6). In contrast, clinically significant IPCa might call for radical prostatectomy or brachytherapy. Thus, it is crucial to identify predictive factors for IPCa before BPH surgery to aid in preoperative counseling and patient expectations management.

Previously reported IPCa risk predictions mainly involve the following indicators: age (5, 7, 8), body mass index (BMI) (9), preoperative prostate-specific antigen (pre-PSA) (10), preoperative prostate-specific antigen density (pre-PSAD) (5), baseline prostate volume (PV) (8), resected prostate weight (11), abnormal digital rectal examination (DRE) findings (7, 12), and preoperative treatment with 5-alpha reductase inhibitors (5αRIs) (11). The conclusions remain controversial and inconsistent, despite previous studies exploring the correlation between the aforementioned factors and IPCa. For example, Guo et al. (8) reported that smaller PV and older age could independently predict an increasing risk for IPCa after BPH surgery, while Porto et al. (13) concluded that PV and age were not significantly related to IPCa.

In 2022, Guo and colleagues (14) conducted a meta-analysis that only investigated the correlation between IPCa and age, PSA, and PV. However, the latest literature they included in their meta-analysis was published in 2018. Many newly published papers focused on the correlation between IPCa and relevant risk factors, reporting different conclusions in recent years (8, 9, 12, 13, 15–23). Thus, we conducted this updated systematic review and meta-analysis with the purpose of finding more evidence to identify the clinical predictors of IPCa after surgery for presumed BPH.

## Materials and methods

We followed the Preferred Reporting Items for Systemic Reviews and Meta-analyses (PRISMA) guidelines to report this systematic review and meta-analysis (24). Additionally, registration of this systematic review and meta-analysis was completed at the International Prospective Register of Systematic Reviews (reference number: CRD42025631346).

### Search strategy

The literature was comprehensively searched using PubMed, Web of Science, Embase, and Cochrane databases in December 2024. Using a combination of Medical Subject Headings and keywords, the search terms included: “incidental,” “prostate cancer,” “risk factors,” “surgery,” and “benign prostatic hyperplasia.” Retrieval was limited to English literature. The data presented in this paper originated from the original article and has received prior ethical approval. Hence, ethical approval was not required for our research, and all analyses were conducted based on previously published studies.

### Inclusion and exclusion criteria

The following PICO guided eligibility screening of studies:

1. participants: men undergoing surgery for presumed BPH;
2. intervention: clinical predictors associated with IPCa (such as BMI, age, pre-PSA, pre-PSAD, baseline PV, etc.);
3. comparisons: this would involve comparing patients with IPCa detected after surgery to those without; For example, comparing BMI, age, pre-PSA, pre-PSAD, and baseline PV levels between the two groups;
4. outcomes: histologically confirmed IPCa after surgery as the outcome of interest.

Studies were excluded if they:

1. were review articles, non-original articles, case reports, editorials, and comments;
2. did not assess the association between the occurrence of IPCa after BPH surgery and clinical factors;
3. did not provide sufficient relevant data to obtain standardized mean differences (SMDs) and odds ratios (ORs), along with their 95% confidence intervals (CIs).

Two researchers (C Yin and Y Wu) independently searched the literature and resolved any conflicts through discussion.

### Data extraction and quality assessment

The data extraction was independently performed by the same two investigators (C Yin and Y Wu), while another researcher (X Chen) verified the accuracy of all extractions. The primary information we extracted from the included studies was as follows: publication information (first author, publication year, study period, geographical region, and study design), clinical information (sample size, surgical methods, BMI, age, pre-PSA, pre-PSAD, baseline PV, resected prostate weight, preoperative treatment with 5αRIs, family history of PCa, DRE findings, transrectal ultrasonography (TRUS) findings, preoperative negative prostate biopsy, smoking history, and magnetic resonance imaging (MRI) findings), and comorbidities (history of hypertension, history of diabetes, and history of dyslipidemia). Each study included in this meta-analysis was independently assessed for quality by two reviewers (Y Wang and H Yang) using the Newcastle-Ottawa quality assessment scale (NOS) (25). The NOS encompasses three dimensions, with a total score of nine stars. The three dimensions include selection with four items, comparability with one item, and exposure/outcome with three items. Each item represents 1 point, except for comparability, which represents 2 points. A total of 1 – 3 stars indicates low quality, 4 – 6 stars indicate medium quality, and 7 – 9 stars indicate high quality.

### Statistical analysis

This meta-analysis used pooled SMDs and ORs with their 95% CIs to describe the correlation between relevant risk factors and the occurrence of IPCa after BPH surgery. The occurrence of IPCa was closely associated with risk factors if the pooled SMD was greater than 0 or the OR was greater than 1. The assessment of heterogeneity among studies involved Cochrane’s Q and I² tests (26). An I^2^ value greater than 50% or a *P*_heterogeneity_ less than 0.05 indicated significant heterogeneity, and a DerSimonian and Laird random-effects (RE) model was utilized. In situations where the I^2^ value was below 50% and *P*_heterogeneity_ exceeded 0.05, the fixed-effects (FE) model was applied. The reasons for heterogeneity were explored through subgroup analysis and meta-regression analysis. A sensitivity analysis was conducted by omitting one study at a time to test the reliability of the findings. Begg’s funnel plots and Egger’s test were employed to assess potential publication bias. All statistical analyses were carried out using STATA version 18.0.

## Results

### Literature search

**Figure 1:**
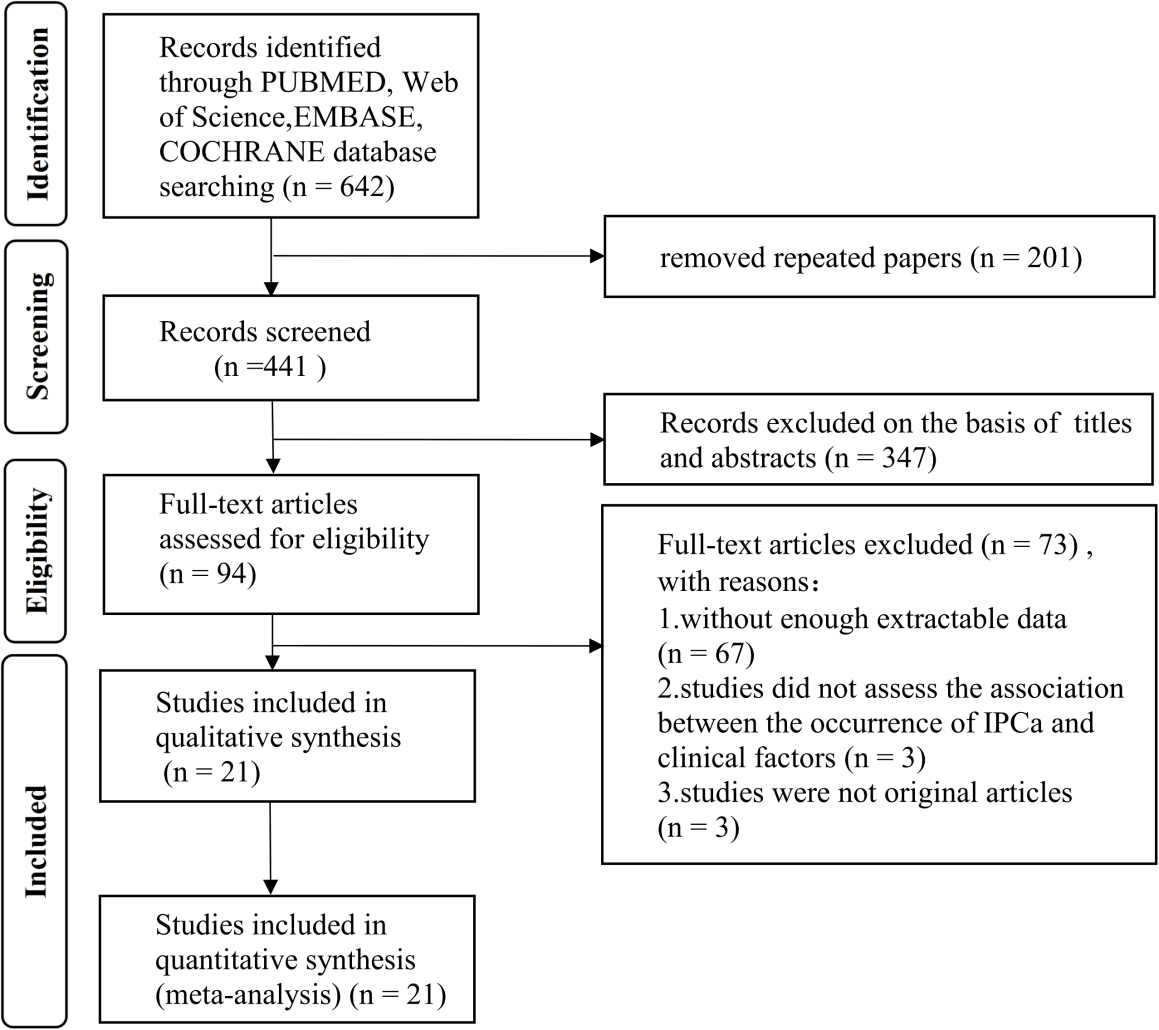
Flow diagram of literature searches according to the Preferred Reporting Items for Systematic Reviews and Meta-analyses guidelines.

**Figure 1** presents the PRISMA flow diagram, which details the literature selection process. A total of 642 records were retrieved from the electronic database based on the search criteria. After removing duplicate records, 441 entries remained. Following the review of titles and abstracts, 347 records were removed. Subsequently, we conducted a thorough analysis of the full-text and excluded 73 records, with 67 papers not having enough extractable data, three studies did not assess the association between the occurrence of IPCa and clinical factors, and three studies were not original articles. This meta-analysis ultimately incorporated 21 eligible studies (7–9, 12, 13, 15–23, 27–33) containing data for 10,842 patients published between 2006 and 2024.

### Features of the Included Studies

The studies we included were of a retrospective design. The main traits of the included studies were summarized and presented in **Table 1**. Altogether, there were 10,842 patients, with sample sizes ranging from 84 to 1,613. After BPH surgery, 957 patients were histopathologically diagnosed with IPCa, accounting for 8.83% of the entire sample. The geographical distribution of the studies included four in the USA, three in Korea, three in Japan, two in China, two in Canada, two in Italy, as well as one each in France, Turkey, Brazil, Somalia, and Tanzania. Regarding surgical methods, ten studies employed holmium laser enucleation of the prostate (HoLEP), six utilized TURP, two applied TURP/open prostatectomy (OP), one adopted OP, one used TURP/HoLEP/robotic-assisted simple prostatectomy, and one used green laser enucleation of the prostate/OP. The range of quality scores determined by the NOS was from 6 to 9 (**Supplementary Table 1**).

**Table 1:** The main characteristics of studies included in this meta-analysis.

First author	Year/Period	Country	Methods	Patients, *n*		Age, years		pre-PSA, ng/ml		Baseline PV, ml	
				IPCa	BPH	IPCa	BPH	IPCa	BPH	IPCa	BPH
Li et al. (15)	2024	USA	HoLEP	104	707	Median (IQR)	Median (IQR)	Median (IQR)	Median (IQR)	Median (IQR)	Median (IQR)
	(2021-2022)					73 (67 – 77)	71 (65 – 77)	4.2 (2.3 – 6.9)	4.2 (2.0 – 7.4)	94 (60 – 138)	108 (73 – 150)
Bendari et al. (16)	2024	USA	TURP	65	66	NA	Median (IQR)	NA	Median (IQR)	NA	NA
	(2019-2023)						71 (11.5)		3 (5)		
Porto et al. (13)	2024	USA	HoLEP	40	377	NA	Mean ± SD	NA	Median (IQR)	NA	Median (IQR)
	(2017-2022)						69.0 ± 8.4		4.4 (2.2 – 8.2)		105 (74 – 157)
Mohamed et al. (17)	2023	Somalia	TURP/OP	95	443	Mean ± SD	Mean ± SD	Mean ± SD	Mean ± SD	Mean ± SD	Mean ± SD
	(2017-2022)					74.0 ± 10.9	71.3 ± 10.8	4.64 ± 3.53	3.41 ± 2.82	70.9 ± 16.5	81.9 ± 20.8
Yang et al. (18)	2022	China	TURP/RASP/HoLEP	57	238	Mean ± SD	Mean ± SD	Median (IQR)	Median (IQR)	Median (IQR)	Median (IQR)
	(2015-2017)					69.14 ± 5.94	62.52 ± 4.46	12.18 (5.39)	5.35 (4.30)	47.18 (26.29)	69.48 (22.36)
Guo et al. (8)	2022	China	TURP	12	241	Mean ± SD	Mean ± SD	Mean ± SD	Mean ± SD	Mean ± SD	Mean ± SD
	(2016-2021)					74.4 ± 6.10	69.6 ± 7.06	22.8 ± 22.34	17.1 ± 14.69	67.7 ± 23.28	90.4 ± 50.02
Banno et al. (19)	2022	Japan	HoLEP	49	563	Median (IQR)	Median (IQR)	Median (IQR)	Median (IQR)	Median (IQR)	Median (IQR)
	(2013-2021)					74 (69 – 78)	73 (67 – 77)	6.5 (3.9 – 10.5)	5.8 (3.0 – 10.2)	55.4 (43.0 – 80.2)	60.2 (44.2 – 79.5)
Kizilkan et al. (12)	2022	Turkey	OP	24	406	Mean ± SD	Mean ± SD	Median (Range)	Median (Range)	Median (Range)	Median (Range)
	(2010-2019)					73.67 ± 6.96	69.22 ± 7.97	7.63 (0.92 – 48.77)	7.47 (0.18 – 31.15)	97 (63 – 280)	95 (61 – 450)
Porcaro et al. (9)	2021	Italy	TURP	30	424	Mean ± SD	Mean ± SD	Mean ± SD	Mean ± SD	NA	NA
	(2016-2018)					71.9 ± 8.4	68.9 ± 8.4	3.4 ± 2.1	3.4 ± 4.7		
Porcaro et al. (20)	2021	Italy	TURP	18	371	NA	NA	NA	NA	NA	NA
	(2017-2019)										
Tominaga et al. (21)	2019	Japan	HoLEP	25	393	Mean ± SD	Mean ± SD	Mean ± SD	Mean ± SD	Mean ± SD	Mean ± SD
	(2008-2016)					75.5 ± 7.3	71.7 ± 8.2	5.82 ± 4.61	5.29 ± 6.76	48.2 ± 24.8	46.9 ± 27.0
Kim et al. (22)	2019	Korea	HoLEP	20	153	Median (IQR)	Median (IQR)	Median (IQR)	Median (IQR)	Median (IQR)	Median (IQR)
	(2009-2015)					70.26 (64.14 – 74.95)	70.84 (65.84 – 74.50)	7.44 (5.86 – 9.03)	4.85 (4.21 – 6.32)	45.95 (31.70 – 68.58)	58.80 (46.50 – 84.25)
Misraï et al. (23)	2019	France	GreenLEP/OP	37	365	Median (IQR)	Median (IQR)	Median (IQR)	Median (IQR)	Median (IQR)	Median (IQR)
	(2005-2018)					72 (63 – 77)	70 (65 – 76)	8.1 (4.9 – 12)	6.8 (4.1 – 10)	100 (100 – 130)	110 (100 – 140)
Gunda et al. (27)	2018	Tanzania	TURP	33	119	Mean ± SD	Mean ± SD	Mean ± SD	Mean ± SD	Mean ± SD	Mean ± SD
	(2015)					71 ± 8.0	68 ± 9.6	34.1 ± 26.7	13.4 ± 18.8	83.4 ± 51.1	95.20 ± 61.9
Ohwaki et al. (28)	2017	Japan	HoLEP	41	613	Mean ± SD	Mean ± SD	Median (IQR)	Median (IQR)	Median (IQR)	Median (IQR)
	(2008-2014)					70 ± 7	70 ± 7	6.71 (5.12 – 13.00)	6.10 (3.59 – 10.50)	81 (54 – 100)	66 (50 – 90)
Elkoushy et al. (29)	2015	Canada	HoLEP	70	1172	Mean ± SD	Mean ± SD	Mean ± SD	Mean ± SD	Mean ± SD	Mean ± SD
	(1998-2014)					75.8 ± 8.7	71.9 ± 8.1	13.6 ± 15.7	6.14 ± 8.37	85.97 ± 46.2	95.23 ± 50.0
Bhojani et al. (30)	2015	Canada	HoLEP	103	1169	Mean ± SD	Mean ± SD	Mean (Range)	Mean (Range)	Mean (Range)	Mean (Range)
	(1998-2013)					74.5 ± 9	70 ± 8	9.8 (0.13 – 61)	7.0 (0.04 – 121)	85.2 (23 – 231)	101.4 (9 – 391)
Kim et al. (31)	2014	Korea	HoLEP	15	269	NA	NA	Mean ± SD	Mean ± SD	NA	NA
	(2008-2011)							1.63 ± 0.89	1.70 ± 0.92		
Yoo et al. (32)	2012	Korea	TURP	78	1535	Mean ± SD	Mean ± SD	Mean ± SD	Mean ± SD	Mean ± SD	Mean ± SD
	(2004-2008)					72.4 ± 7.5	71.1 ± 7.6	6.9 ± 5.4	4.7 ± 4.2	54.4 ± 31.5	59.5 ± 30.5
Nunez et al. (33)	2011	USA	HoLEP	28	56	Median (Range)	Median (Range)	Median (Range)	Median (Range)	Median (Range)	Median (Range)
	(2007-2010)					73.0 (54.0 – 87.0)	73.0 (54.0 – 86.0)	3.3 (0.7 – 56.8)	3.3 (0.7 – 13.3)	71.2 (26.9 – 140.0)	68.1 (27.4 – 145.0)
Antunes et al. (7)	2006	Brazil	TURP/OP	13	205	Mean ± SD	Mean ± SD	NA	NA	Mean ± SD	Mean ± SD
	NA					73.9 ± 11.2	68.0 ± 7.4			72 ± 28.8	65 ± 33.9

NA, data not applicable; SD, standard deviation; IQR, interquartile range; pre-PSA, preoperative prostate-specific antigen; PV, prostate volume; IPCa, incidental prostate cancer; BPH, benign prostatic hyperplasia; HoLEP, Holmium laser enucleation of the prostate; TURP, transurethral resection of the prostate; RASP, robotic-assisted simple prostatectomy; OP, open prostatectomy; GreenLEP, green laser enucleation of the prostate.

### Meta-Analysis

**Figure 2:**
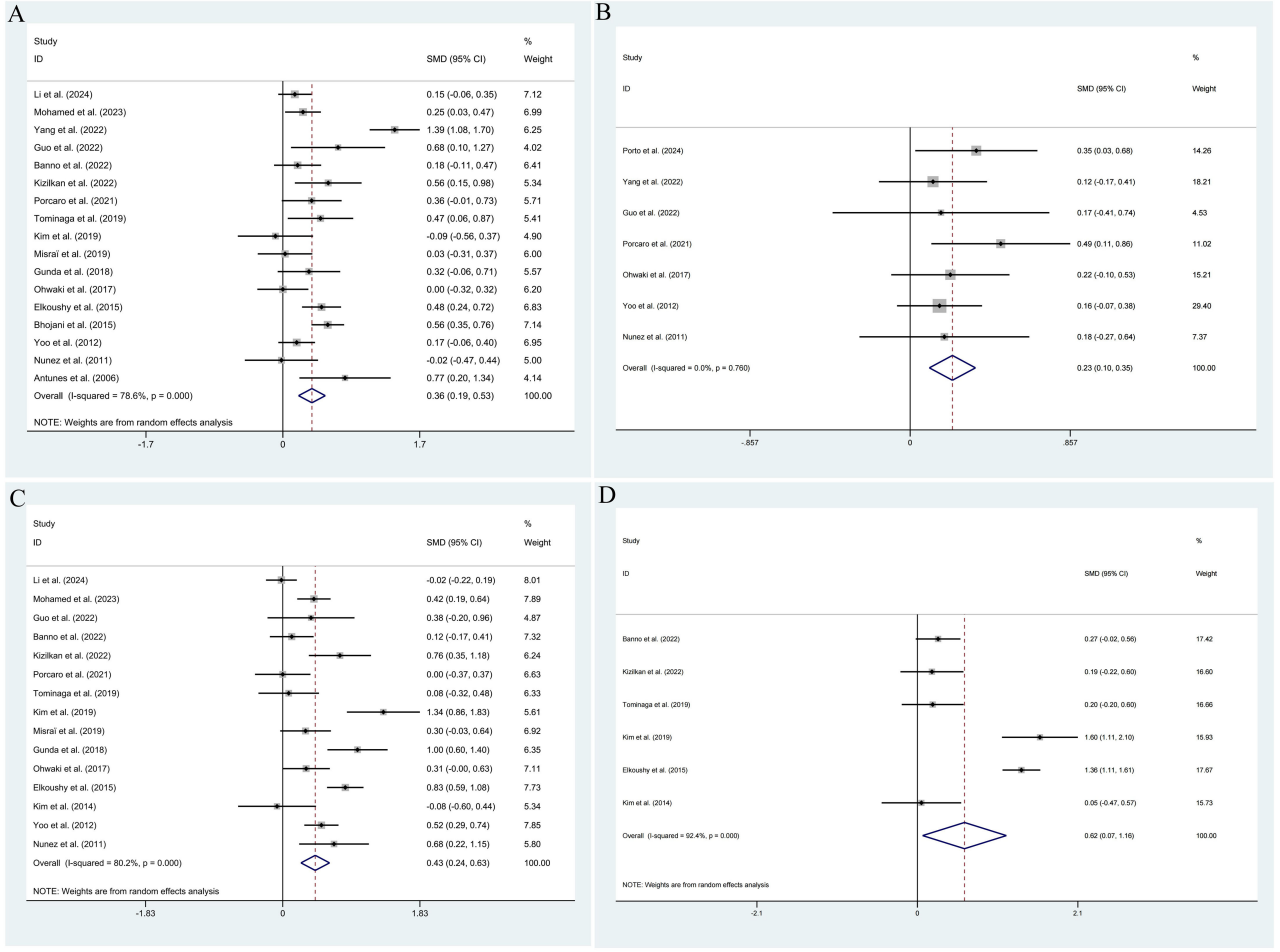
Forest plots of studies assessing the predictors for age **(A)**, BMI **(B)**, pre-PSA **(C)**, and pre-PSAD **(D)** with IPCa risk.

**Figure 3:**
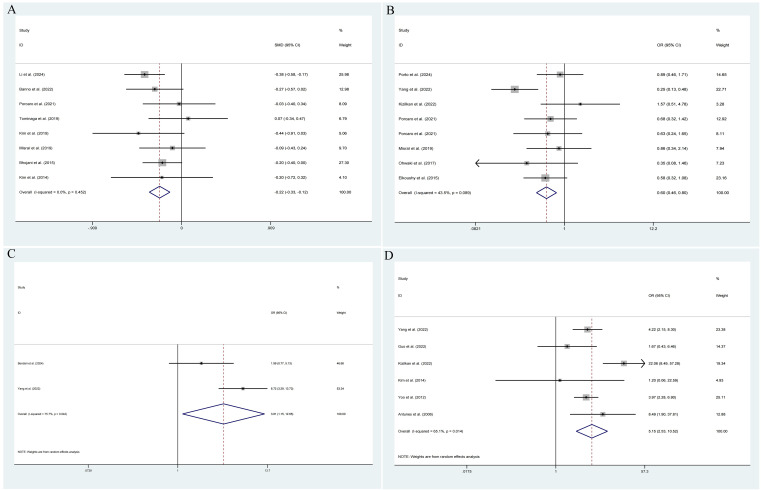
Forest plots of studies assessing the predictors for resected prostate weight **(A)**, preoperative treatment with 5αRIs **(B)**, family history **(C)**, and DRE findings **(D)** with IPCa risk.

**Figure 4:**
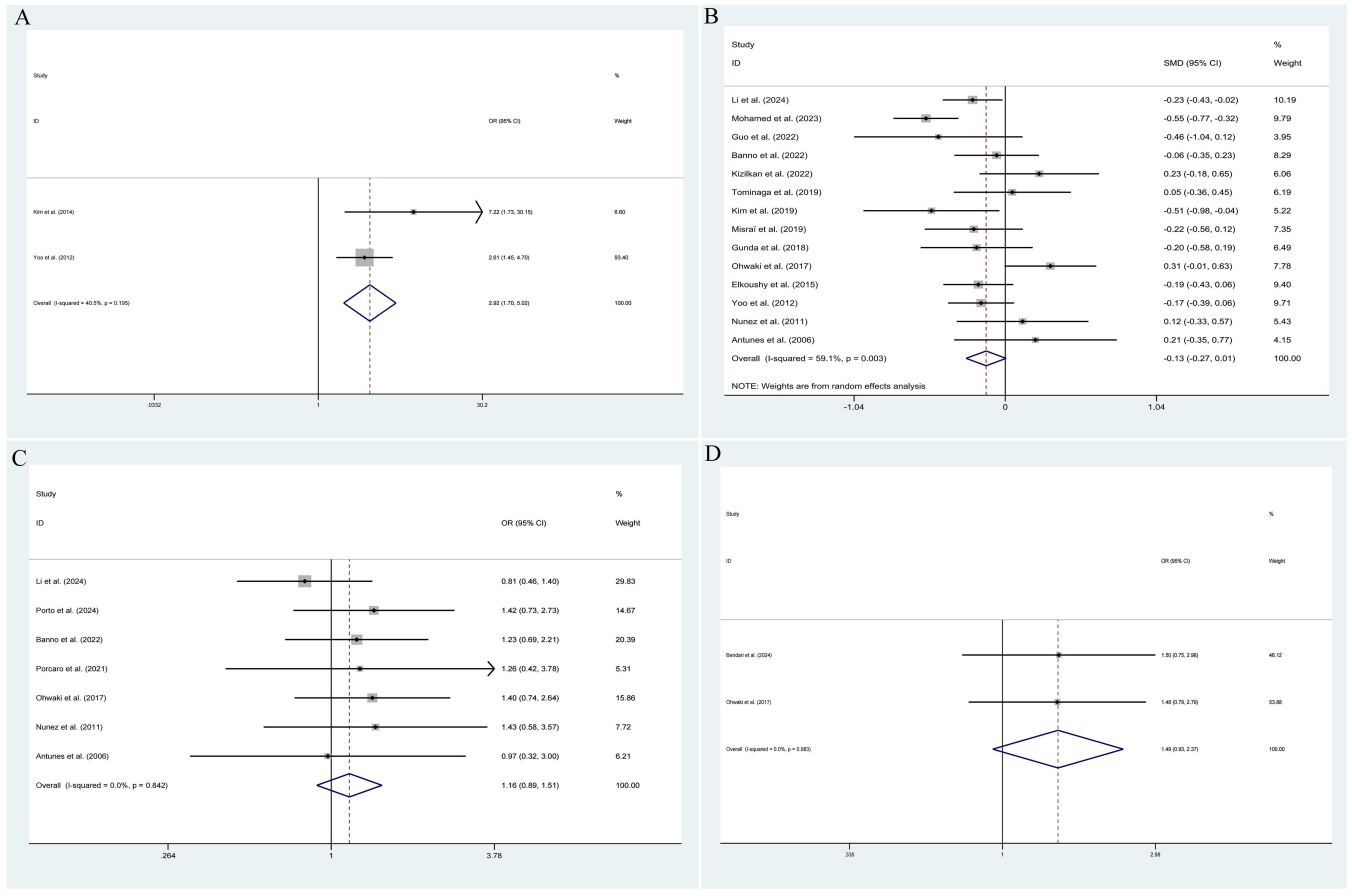
Forest plots of studies assessing the predictors for TRUS findings **(A)**, baseline PV **(B)**, preoperative negative prostate biopsy **(C)**, and smoking history **(D)** with IPCa risk.

**Figure 5:**
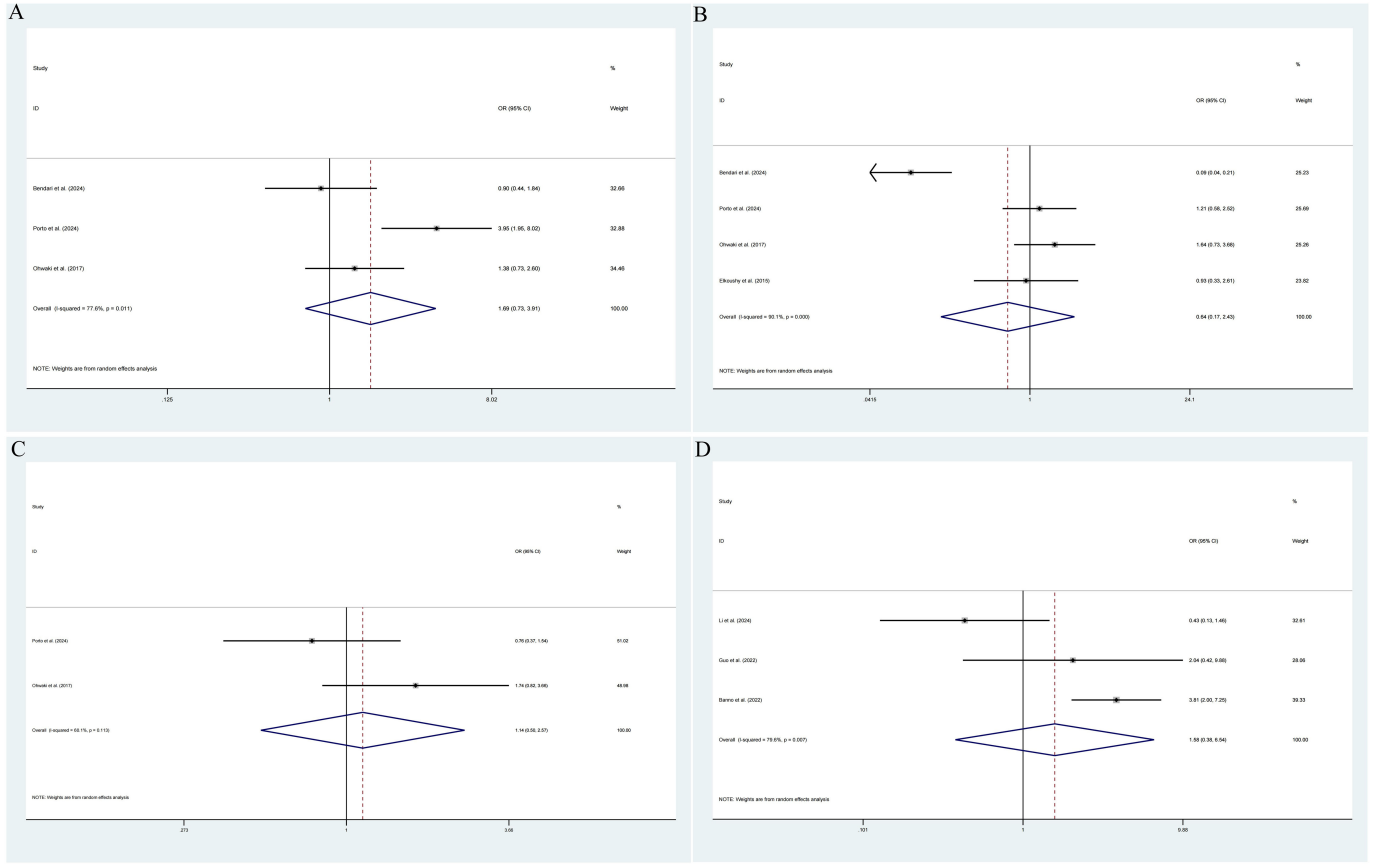
Forest plots of studies assessing the predictors for history of hypertension **(A)**, history of diabetes **(B)**, history of dyslipidemia **(C)**, and MRI findings **(D)** with IPCa risk.

The pooled results demonstrated that the occurrence of IPCa was significantly associated with age (RE model, pooled SMD = 0.36; 95% CI: 0.19 – 0.53; *P* < 0.001, **Figure 2A**), BMI (FE model, pooled SMD = 0.23; 95% CI: 0.10 – 0.35; *P* < 0.001, **Figure 2B**), pre-PSA (RE model, pooled SMD = 0.43; 95% CI: 0.24 – 0.63; *P* < 0.001, **Figure 2C**), pre-PSAD (RE model, pooled SMD = 0.62; 95% CI: 0.07 – 1.16; *P* = 0.028, **Figure 2D**), resected prostate weight (FE model, pooled SMD = -0.22; 95% CI: -0.33 – -0.12; *P* < 0.001, **Figure 3A**), preoperative treatment with 5αRIs (yes/no) (FE model, pooled OR = 0.60; 95% CI: 0.46 – 0.80; *P* < 0.001, **Figure 3B**), family history (yes/no) (RE model, pooled OR = 3.81; 95% CI: 1.15 – 12.65; *P* = 0.029, **Figure 3C**), DRE findings (abnormal/normal) (RE model, pooled OR = 5.15; 95% CI: 2.53 – 10.52; *P* < 0.001, **Figure 3D**), and TRUS findings (abnormal/normal) (FE model, pooled OR = 2.92; 95% CI: 1.70 – 5.02; *P* < 0.001, **Figure 4A**). Additionally, we identified that no significant associations existed between the occurrence of IPCa and baseline PV (RE model, pooled SMD = -0.13; 95% CI: -0.27 – 0.01; *P* = 0.060, **Figure 4B**), preoperative negative prostate biopsy (yes/no) (FE model, pooled OR = 1.16; 95% CI: 0.89 – 1.51; *P* = 0.275, **Figure 4C**), smoking history (yes/no) (FE model, pooled OR = 1.49; 95% CI: 0.93 – 2.37; *P* = 0.096, **Figure 4D**), history of hypertension (yes/no) (RE model, pooled OR = 1.69; 95% CI: 0.73 – 3.91; *P* = 0.218, **Figure 5A**), history of diabetes (yes/no) (RE model, pooled OR = 0.64; 95% CI: 0.17 – 2.43; *P* = 0.514, **Figure 5B**), history of dyslipidemia (yes/no) (RE model, pooled OR = 1.14; 95% CI: 0.50 – 2.57; *P* = 0.754, **Figure 5C**), and MRI findings (abnormal/normal) (RE model, pooled OR = 1.58; 95% CI: 0.38 – 6.54; *P* = 0.532, **Figure 5D**).

### Subgroup analysis and meta-regression analysis

Given the relatively small number of studies assessing family history, TRUS findings, smoking history, history of hypertension, history of dyslipidemia, and MRI findings, along with the lack of significant heterogeneity in BMI, resected prostate weight, and preoperative negative prostate biopsy, we only performed subgroup analyses for age, pre-PSA, pre-PSAD, baseline PV, preoperative treatment with 5αRIs, DRE findings, and history of diabetes (**Table 2**). Subgroup analyses were carried out according to the surgical method (HoLEP only vs. other method), the geographical region (Asian vs. non-Asian), year of publication (> 2020 vs. < 2020), and number of patients (> 500 vs. < 500). Subgroup analysis results were generally in agreement with the overall findings. Additionally, a notable decline in heterogeneity was observed in some subgroup analyses, such as those involving more than 500 patients, studies published before 2020, studies carried out in non-Asia, and studies only utilizing the HoLEP technique. To better understand heterogeneity, we performed a meta-regression analysis for several predictors (e.g., age, pre-PSA). For age, we found that the number of patients was a source of heterogeneity (*P* = 0.022), while the surgical method, the geographical region, and the year of publication were not (all *P* > 0.05). Similarly, the number of patients was also a source of heterogeneity for pre-PSA (*P* = 0.023), while the surgical method, the geographical region, and the year of publication were not (all *P* > 0.05).

**Table 2:** The summary and subgroup analysis results for IPCa in this meta-analysis.

Analysis specification	Studies, *n*	Study heterogeneity		Effect	Pooled OR/SMD	*P* Value	Publication bias
		*I²* (%)	*P*_heterogeneity_	model	(95%CI)		*P* for Egger
Age							
Overall	17	78.6	<0.001	Random	0.36 (0.19, 0.53)	<0.001	0.782
Surgical methods							
HoLEP only	8	64.3	0.006	Random	0.25 (0.07, 0.42)	0.006	
Other method	9	84.7	<0.001	Random	0.49 (0.20, 0.78)	0.001	
Geographical region							
Asian	8	87.7	<0.001	Random	0.42 (0.07, 0.77)	0.020	
non-Asian	9	54.1	0.026	Random	0.32 (0.17, 0.46)	<0.001	
Year of publication							
>2020	7	88.0	<0.001	Random	0.50 (0.16, 0.83)	0.004	
<2020	10	61.5	0.005	Random	0.27 (0.10, 0.44)	0.001	
Number of patients							
>500	7	61.8	0.015	Random	0.27 (0.12, 0.41)	<0.001	
<500	10	82.9	<0.001	Random	0.45 (0.13, 0.76)	0.006	
BMI							
Overall	7	0	0.760	Fixed	0.23 (0.10, 0.35)	<0.001	0.523
pre-PSA							
Overall	15	80.2	<0.001	Random	0.43 (0.24, 0.63)	<0.001	0.454
Surgical methods							
HoLEP only	8	86.6	<0.001	Random	0.40 (0.08, 0.72)	0.014	
Other method	7	62.8	0.013	Random	0.48 (0.27, 0.68)	<0.001	
Geographical region							
Asian	8	74.8	<0.001	Random	0.42 (0.16, 0.69)	0.002	
non-Asian	7	86.0	<0.001	Random	0.45 (0.15, 0.75)	0.003	
Year of publication							
>2020	6	70.1	0.005	Random	0.25 (0.02, 0.48)	0.036	
<2020	9	77.5	<0.001	Random	0.56 (0.31, 0.80)	<0.001	
Number of patients							
>500	6	84.5	<0.001	Random	0.36 (0.11, 0.62)	0.005	
<500	9	78.2	<0.001	Random	0.49 (0.19, 0.80)	0.002	
Baseline PV							
Overall	14	59.1	0.003	Random	-0.13 (-0.27, 0.01)	0.060	0.223
Surgical methods							
HoLEP only	7	53.1	0.046	Random	-0.07 (-0.25, 0.10)	0.405	
Other method	7	61.9	0.015	Random	-0.19 (-0.41, 0.02)	0.080	
Geographical region							
Asian	7	56.1	0.033	Random	-0.05 (-0.26, 0.15)	0.614	
non-Asian	7	51.2	0.056	Random	-0.21 (-0.38, -0.05)	0.012	
Year of publication							
>2020	5	71.9	0.007	Random	-0.22 (-0.48, 0.04)	0.096	
<2020	9	40.6	0.097	Fixed	-0.08 (-0.23, 0.07)	0.309	
Number of patients							
>500	6	75.3	0.001	Random	-0.16 (-0.36, 0.04)	0.117	
<500	8	31.5	0.177	Fixed	-0.09 (-0.28, 0.10)	0.335	
pre-PSAD							
Overall	6	92.4	<0.001	Random	0.62 (0.07, 1.16)	0.028	0.462
Year of publication							
>2020	2	0	0.755	Fixed	0.24 (0.01, 0.48)	0.045	
<2020	4	92.8	<0.001	Random	0.81 (0.07, 1.55)	0.032	
Resected prostate weight							
Overall	8	0	0.452	Fixed	-0.22 (-0.33, -0.12)	<0.001	0.360
Preoperative treatment with 5αRIs							
Overall	8	43.5	0.089	Fixed	0.60 (0.46, 0.80)	<0.001	0.597
Surgical methods							
HoLEP only	3	0	0.424	Fixed	0.64 (0.42, 0.98)	0.041	
Other method	5	61.2	0.036	Random	0.57 (0.39, 0.84)	0.004	
Geographical region							
Asian	3	74.4	0.020	Random	0.40 (0.23, 0.70)	0.001	
non-Asian	5	0	0.899	Fixed	0.71 (0.51, 0.98)	0.039	
Year of publication							
>2020	5	64.4	0.024	Random	0.61 (0.43, 0.87)	0.006	
<2020	3	0	0.560	Fixed	0.60 (0.37, 0.96)	0.034	
Number of patients							
>500	2	0	0.511	Fixed	0.53 (0.30, 0.93)	0.026	
<500	6	57.6	0.038	Random	0.64 (0.46, 0.89)	0.007	
Family history							
Overall	2	75.7	0.042	Random	3.81 (1.15, 12.65)	0.029	NA
DRE findings							
Overall	6	65.1	0.014	Random	5.15 (2.53, 10.52)	<0.001	0.975
Year of publication							
>2020	3	83.3	0.002	Random	5.66 (1.45, 22.06)	0.013	
<2020	3	0	0.448	Fixed	4.18 (2.51, 6.97)	<0.001	
TRUS findings							
Overall	2	40.5	0.195	Fixed	2.92 (1.70, 5.02)	<0.001	NA
Preoperative negative prostate biopsy							
Overall	7	0	0.842	Fixed	1.16 (0.89, 1.51)	0.275	0.616
Smoking history							
Overall	2	0	0.983	Fixed	1.49 (0.93, 2.37)	0.096	NA
History of hypertension							
Overall	3	77.6	0.011	Random	1.69 (0.73, 3.91)	0.218	0.900
History of diabetes							
Overall	4	90.1	<0.001	Random	0.64 (0.17, 2.43)	0.514	0.957
Year of publication							
>2020	2	95.2	<0.001	Random	0.34 (0.03, 4.16)	0.398	
<2020	2	0	0.393	Fixed	1.32 (0.70, 2.50)	0.392	
Number of patients							
>500	2	0	0.393	Fixed	1.32 (0.70, 2.50)	0.392	
<500	2	95.2	<0.001	Random	0.34 (0.03, 4.16)	0.398	
History of dyslipidemia							
Overall	2	60.1	0.113	Random	1.14 (0.50, 2.57)	0.754	NA
MRI findings							
Overall	3	79.6	0.007	Random	1.58 (0.38, 6.54)	0.532	0.511

IPCa, incidental prostate cancer; OR, odds ratio; SMD, standardized mean difference; CI, confidence interval; BMI, body mass index; pre-PSA, preoperative prostate-specific antigen; pre-PSAD, preoperative prostate-specific antigen density; PV, prostate volume; NA, data not applicable; HoLEP, Holmium enucleation of the prostate; 5αRIs, 5-alpha reductase inhibitors; DRE, digital rectal examination; TRUS, transrectal ultrasonography; MRI, magnetic resonance imaging.

### Sensitivity analysis and publication bias

We carried out a sensitivity analysis to verify the reliability of the results. The sensitivity analysis validated that the overall findings were unaffected by any single study (**Supplementary Figure 1**). Begg’s funnel plots showed no signs of asymmetry (**Supplementary Figure 2**). Additionally, Egger’s test further confirmed the absence of publication bias for age (*P* = 0.782), BMI (*P* = 0.523), pre-PSA (*P* = 0.454), pre-PSAD (*P* = 0.462), baseline PV (*P* = 0.223), resected prostate weight (*P* = 0.360), preoperative treatment with 5αRIs (*P* = 0.597), DRE findings (*P* = 0.975), preoperative negative prostate biopsy (*P* = 0.616), history of hypertension (*P* = 0.900), history of diabetes (*P* = 0.957), and MRI findings (*P* = 0.511) (**Table 2**).

## Discussion

Based on data from 21 retrospective studies, our study investigated the predictive factors associated with the increasing risk of IPCa following BPH surgery. This meta-analysis included 10,842 patients, of whom 957 were histopathologically diagnosed with IPCa. The IPCa rate after surgery for presumed BPH was 8.83%. Most importantly, our study identified that age, BMI, pre-PSA, pre-PSAD, resected prostate weight, preoperative treatment with 5αRIs, family history, abnormal DRE findings, and abnormal TRUS findings were significantly associated with the occurrence of IPCa. However, there were no significant associations between IPCa and baseline PV, preoperative negative prostate biopsy, smoking history, history of hypertension, history of diabetes, history of dyslipidemia, and abnormal MRI findings. Similar findings were observed in subgroup analyses when the study was stratified by surgical method, geographical region, year of publication, and number of patients. In addition, the sensitivity analysis and publication bias also suggested that our findings were reliable and robust. The rates of IPCa detection mentioned in the published literature vary significantly. The IPCa rate in our study was 8.83%. According to a multicenter study conducted by Anract and colleagues (5), the rate of IPCa was 10.1% in a cohort of 2,452 patients. Elkoushy et al. (29) reported that only 5.64% of the 1,242 patients in their study had IPCa. Yilmaz et al. (34) conducted a literature review and demonstrated that the rate of IPCa varied from 5.64% to 23.3%. The discrepancies can be attributed to several factors, including the amount of prostate tissue resected and the population involved. The IPCa rate may be affected by the PCa screening policy.

Age remains the most frequent risk factor associated with the development of PCa. The risk of PCa tends to increase as men age, potentially as a consequence of the aging process. Several studies have demonstrated that increasing age could independently predict IPCa following BPH surgery (5, 7, 8, 17, 29, 30, 35). However, Porto et al. (13) reported no significant association between age and IPCa. Guo et al. (14) performed a meta-analysis of eight studies and concluded that there was no significant association between increasing age and IPCa. Herein, we conducted an updated meta-analysis of twenty-one studies to provide more accurate evidence and identified that increasing age was significantly associated with an increasing risk of IPCa.

Vidal and colleagues (36) claimed that BMI might play a key role in the pathogenesis of PCa. Porcaro et al. (9) demonstrated that BMI was significantly associated with IPCa after BPH surgery. Several mechanisms have previously been identified through which obesity promotes the progression of PCa (37). These factors include the metabolic impact resulting from the deregulation of the insulin/insulin-like growth factor-1 axis, reduced testosterone levels in obese males, and the paracrine influence of hypertrophic adipocytes surrounding tumors (38–40). A higher BMI might make it harder to detect PCa and is associated with more aggressive PCa (41). However, Guo et al. (8) found no significant association between BMI and IPCa in the multivariate analysis. Our pooled results confirmed that a higher BMI was significantly associated with an increasing risk of IPCa.

Several studies have reported a significant correlation between PSA and IPCa (16–18, 27, 32). On the contrary, Porto et al. (13) demonstrated that baseline PSA was not associated with IPCa. There are several possible reasons why preoperative PSA levels may not predict the occurrence of IPCa. First, baseline PSA levels in patients with BPH could be elevated due to urinary retention and the use of urinary catheters. Second, PSA screening before surgery lowers the risk of detecting IPCa. The ratio of serum total PSA to PV is known as PSAD. Higher PSAD values will increase PSA release per unit volume, with PCa cells more strongly disrupting the normal acinar structure, which often indicates a more aggressive and malignant tumor. Anract et al. (5) demonstrated that PSAD could independently predict IPCa. Similarly, Elkoushy et al. (29) found that pre-PSAD could be independently predictive of IPCa after HoLEP. Contrary to the above results, Banno and colleagues (19) reported no significant association between PSAD and IPCa. Our pooled results validated that higher pre-PSA and pre-PSAD were significantly associated with an increasing risk of IPCa.

Currently, the correlation between PV and IPCa remains controversial. Several studies have reported a significant correlation between a smaller PV and an increasing risk of IPCa (8, 17, 30). Moolupuri and colleagues (42) conducted a systematic review and meta-analysis and unveiled that 90% of included studies (27/30) exhibited significant evidence supporting the hypothesis that larger PV may be protective of PCa. Similarly, Al-Khalil et al. (43) demonstrated that there was an association between PV and the incidence and aggressiveness of PCa. The larger the PV, the lower the positive biopsy rate for PCa and the lower the Gleason score. Additionally, Barone et al. (44) reported that elevated BMI was related to larger PV, which may have significant implications for the diagnosis, management, and treatment of BPH and PCa. Several possible explanations are detailed below. First, since the growth of PV depends on androgen levels, serum androgen levels may be a causative factor. Schatzl et al. (45) identified an association between high Gleason scores in PCa patients and reduced testosterone levels. Thus, a smaller PV in patients might be associated with a more aggressive PCa. Second, the growth of the transition zone related to BPH may limit the epithelial cells in the peripheral zone, resulting in their atrophy or apoptosis, which could lower the risk of tumor development in the transition zone (46). However, Porto and colleagues (13) reported no significant association between PV and IPCa. The differences in ultrasound diagnostic methods could be the reason for the inconsistent results. The PV measured by TRUS may differ from that of abdominal ultrasonography. This meta-analysis, which included 15 eligible studies, confirmed no significant association between PV and IPCa, consistent with the results of a previous meta-analysis (14).

A large randomized controlled trial has well established that the use of 5αRIs lowers the risk of PCa (47). Cheng et al. (11) found that preoperative treatment with 5αRIs was significantly associated with the occurrence of IPCa. However, Porcaro et al. (9) reported no significant association between preoperative treatment with 5αRIs and IPCa. The uncertain duration of drug consumption and the small sample size may account for the inconsistent findings. Liu et al. (48) retrospectively reviewed 49,206 patients who underwent BPH surgery, comparing the resected prostate weight with the incidental findings of PCa. They concluded that a higher occurrence of IPCa was observed in presumed BPH patients with a smaller resected prostate weight during TURP. Mohamed and colleagues (17) found a similar association. We speculate that larger-weight specimens may increase pathologists’ difficulty in detecting cancer. However, Misraï et al. (23) reported no significant association between resected prostate weight and IPCa. Herein, this meta-analysis identified that no preoperative treatment with 5αRIs and a smaller resected prostate weight were significantly associated with an increasing risk of IPCa.

Several studies have reported a significant correlation between abnormal DRE findings and IPCa (7, 12, 32). Kizilkan et al. (12) recommended that if a DRE exhibits abnormal results before BPH surgery, the risk of PCa should be seriously assessed, and extra diagnostic tests, including multiparametric MRI and targeted biopsies, should be integrated into the evaluation strategy. However, Yang et al. (18) found that abnormal DRE findings were not significantly associated with IPCa in the multivariate analysis. Kim et al. (31) reported that hypoechoic lesions on TRUS could independently predict IPCa. The study by Shim et al. (49) indicated that patients with only a hypoechoic lesion on TRUS, without high PSA or abnormal DRE, had an 11.5% rate of PCa detection. Onur et al. (50) reported that per-core cancer detection rate of hypoechoic lesions was 9.3% and that prostates with hypoechoic lesions are inclined to have cancers even though the lesions may not contain the tumor. These results indicate that a hypoechoic lesion on TRUS is an important risk factor for PCa. For patients with a normal PSA and negative DRE but who have a hypoechoic lesion on TRUS, a prostate biopsy should be considered prior to BPH surgery. Our pooled results confirmed that abnormal DRE findings, TRUS findings, and family history were significantly associated with an increasing risk of IPCa.

Nowadays, the correlation between preoperative negative prostate biopsy and IPCa remains controversial and inconclusive. Capogrosso and colleagues (2) demonstrated that a preoperative negative prostate biopsy was significantly associated with a reduced risk of IPCa diagnosis following BPH surgery (OR = 0.29, *P* = 0.007). A negative biopsy result before surgery can rule out some PCa cases, thus reducing the risk of IPCa after surgery. Conversely, Kim et al. (31) reported no significant association between preoperative negative prostate biopsy and IPCa. Despite prior biopsies, the un-biopsied transition zone is thought to be a major cause of ongoing PCa risk. The prostate biopsy mainly focuses on the peripheral zone. Scholars have proposed that TURP was essential to exclude the possibility of transition zone PCa in patients who previously had a negative prostate biopsy (51, 52). According to Puppo et al. (51), for patients with repeated negative prostate biopsies, the combination of peripheral zone biopsy and TURP could increase the PCa detection rate to 57%. Herein, this meta-analysis demonstrated that no significant associations existed between IPCa and preoperative negative prostate biopsy. The use of multiparametric MRI has been shown to boost the accuracy of PCa diagnosis in the transition zone (53, 54). Banno and colleagues (19) found that abnormal MRI findings could independently predict IPCa after HoLEP. Massanova et al. (55) retrospectively analyzed 630 patients who underwent transrectal systematic prostate biopsy following multiparametric MRI and demonstrated that the Prostate Imaging Reporting and Data System (PI-RADS) version 2 score and pre-PSAD were independent predictors of PCa and clinically significant PCa. However, Guo et al. (8) reported no significant association between MRI findings and IPCa in the multivariate logistic regression analyses (*P* = 0.637). Similarly, Li et al. (15) reported that PI-RADS was not a significant predictor of IPCa or clinically significant IPCa detection, as most patients with PI-RADS 3 – 5 undergo preoperative biopsy, and demonstrates that MRI fusion biopsy has high negative predictive value for IPCa and clinically significant IPCa (94% and 97%, respectively). Our pooled results confirmed negative results.

The association between comorbidities and IPCa is not well-illuminated. Several studies have demonstrated a negative correlation, significant or insignificant, between diabetes and PCa (56, 57). In contrast, according to Leitzmann et al. (58), diabetic men with a BMI below 25 kg/m^2^ had an increased risk of aggressive PCa. Ohwaki and colleagues (28) reported that hypertension, diabetes, and dyslipidemia could not independently predict IPCa. However, they found a significant association between high-risk IPCa and diabetes. Similarly, Porto et al. (13) demonstrated that hypertension, diabetes, and dyslipidemia were not significantly associated with an increasing risk of IPCa. However, they found that hypertension was significantly associated with grade group 1 PCa. Our pooled results confirmed no significant association between comorbidities and IPCa.

To our knowledge, this study is the most comprehensive investigation of the association between clinical information and IPCa. Our research has led to some meaningful conclusions. In several aspects, our meta-analysis displayed critical advantages. First, a pooled SMD/OR was used to compare the difference between IPCa and non-IPCa in patients following BPH surgery. Second, our results are more reliable than those from a single study because we include a large number of patients from various geographical regions. Third, although a previous meta-analysis explored the association between IPCa and age, PSA, and PV, it included only eight eligible studies, and the most recent literature was published in 2018. Herein, our updated meta-analysis analyzed the relationship between more clinical factors and IPCa, and we included a lot of newly published literature. Based on our results, we could provide robust and reliable evidence for predictors of IPCa after BPH surgery.

Recent research has focused on the correlation between IPCa and clinical parameters to determine the value of predictors. The critical question is how to apply these results to clinical practice, such as risk stratification and treatment decisions for patients. For patients with clinically insignificant IPCa, active surveillance was recommended by most international guidelines. In contrast, clinically significant IPCa might call for radical prostatectomy or brachytherapy. Radical prostatectomy remains a viable option following endoscopic enucleation in high-volume centers; however, the 1-year continence rate is reported to be significantly worse than in patients who have not had previous surgery (59). Challenges in dissection might increase the likelihood of erectile dysfunction after surgery and the risk of complications during the operation. Research indicated that external beam radiation therapy posed a low risk of complications for this specific population (60). Other patients may have been good candidates for focal therapy. As far as we know, focal therapy protocols typically do not include patients who have had previous BPH surgery due to challenges in targeting lesions in the remaining peripheral zone (61). These data suggest that a clinically significant IPCa diagnosis will influence patient management and treatment decisions. In a previous study, Anract et al. (5) constructed a risk stratification model for the likelihood of IPCa in patients undergoing surgery for BPH. They assessed two factors: age > 72 and PSAD > 0.1 ng/ml/cm^3^. According to the decision tree, patients with a PSAD of less than 0.1 ng/ml/cm^3^ had a low risk of any IPCa (7.4%). Similarly, the risk of clinically significant IPCa was also low in this population (0.4%). Patients with PSAD exceeding 0.1 ng/ml/cm^3^ had a 23.4% risk of IPCa and a 12.4% risk of clinically significant IPCa. Among those patients, the likelihood of IPCa was 15.1% for patients below 72 years and 35.4% for those above 72 years. Using the same age cutoff for this population, the risk of clinically significant IPCa elevated from 8.4% to 18.2%. Moreover, several studies focused on PSAD and PI-RADS scores. When the PSAD and PI-RADS scores were ≤ 0.15 ng/ml/cm^3^ and 2, respectively, the clinically significant PCa detection rate was 5.6%, whereas a PSAD of > 0.45 ng/ml/cm^3^ and PI-RADS score of 5 yielded an 82.1% detection rate (62, 63). Additionally, Sakamoto et al. (64) assessed three factors, including age > 75, PV ≤ 50 cc, and the absence of preoperative prostate biopsy despite PSA ≥ 4 ng/ml. In patients who had 2 or 3 of these risk factors, IPCa and clinically significant IPCa were observed in 25% to 50% and 16% to 25% cases, respectively. Thus, combining these predictors helps provide actionable guidance for patient counseling or treatment strategies.

Most IPCa were clinically insignificant PCa (e.g., International Society of Urological Pathology grade group < 2 and < 5% of tissue involved with PCa). These types of tumors are low risk and, even without treatment, generally do not pose a threat to the patient’s life. Active surveillance was recommended by most international guidelines. If the tumor progresses during monitoring, further treatment is considered. By accurately detecting this type of clinically insignificant IPCa, unnecessary intervention measures such as radical prostatectomy and radiotherapy can be avoided for patients, and side effects and complications caused by treatment can be reduced. Avoiding the overtreatment of clinically insignificant IPCa can save a lot of medical resources, including human, material, and financial resources, so that medical resources can be more rationally allocated to patients who really need treatment. Additionally, accurate detection of clinically insignificant IPCa can avoid unnecessary anxiety and psychological burden for patients.

However, we recognize that our study has certain limitations. First, because the studies included were retrospective, our study was more prone to recall and selection biases. Second, we excluded non-English studies and grey literature, which increased the potential for selection bias in our research. Nussbaumer-Streit et al. (65) conducted a meta-epidemiological study and reported that excluding non-English publications from evidence syntheses did not change conclusions. In contrast, the exclusion of grey literature from meta-analyses may increase the risk of publication bias, reduce the comprehensiveness of the evidence, and limit the extrapolation of results. Mcauley and colleagues (66) demonstrated that excluding grey literature in meta-analyses can cause exaggerated estimates of intervention effectiveness. They recommended that meta-analysis should attempt to identify, retrieve, and include all reports, grey and published, that meet predefined inclusion criteria. Third, acute urinary retention or indwelling catheters transiently elevate PSA, confounding preoperative risk stratification. None of the included studies in this meta-analysis adjusted for these factors, potentially inflating PSA’s predictive value. Future studies should standardize PSA measurement timing (e.g., post-catheter removal). Fourth, this meta-analysis only assessed predictors of IPCa and did not adequately distinguish between clinically insignificant and significant IPCa, limiting their utility to guide preoperative and postoperative decision-making and management. Finally, patients’ clinical stage (TNM staging) data was not evaluated because only a few studies have reported this information.

## Conclusion

This meta-analysis revealed that age, BMI, pre-PSA, pre-PSAD, resected prostate weight, preoperative treatment with 5αRIs, family history, DRE findings, and TRUS findings were independent factors predicting IPCa following BPH surgery. Before BPH surgery, factors such as age, BMI, pre-PSA, and pre-PSAD should be considered to assess the risk of IPCa. For high-risk patients, more detailed imaging and needle biopsy are recommended before surgery to avoid missed diagnosis. Patients with a family history of PCa are at high risk for IPCa. For such patients, intensive monitoring before and after surgery, and genetic testing if necessary to assess genetic risk. In the future, more large-scale and well-designed studies are needed to validate our results further.

## Data Availability

The original contributions presented in the study are included in the article/**Supplementary Material**. Further inquiries can be directed to the corresponding author.
