# Supplementary material for: Predictive factors of incidental prostate cancer in patients undergoing surgery for presumed benign prostatic hyperplasia: an updated systematic review and meta-analysis
Source: Front Oncol. 2025 Feb 27;15:1561675. doi: 10.3389/fonc.2025.1561675 (PMC11903258; doi:10.3389/fonc.2025.1561675)
Supplement: Supplementary file 1 [file Table1.docx]

**Table S1. Quality assessment of cohort studies included in this meta-analysis**

| **Study** | **Representativeness of the exposed cohort** | **Selection of the unexposed cohort** | **Ascertainment of exposure** | **Outcome of interest not present at start of study** | **Control for important factor or additional factor** | **Outcome assessment** | **Follow-up long enough for outcomes to occur** | **Adequacy of follow-up of cohort** | **Total quality scores** |
| --- | --- | --- | --- | --- | --- | --- | --- | --- | --- |
| **Li et al.** | ★ | ★ | ★ | ★ | ★ | ★ | ★ | ★ | 8 |
| **Bendari et al.** | ★ | ★ | ★ | ★ | ★ | ★ | ★ | — | 7 |
| **Porto et al.** | ★ | ★ | ★ | ★ | ★ | ★ | ★ | ★ | 8 |
| **Mohamed et al.** | ★ | ★ | ★ | ★ | ★★ | ★ | ★ | ★ | 9 |
| **Yang et al.** | ★ | ★ | ★ | ★ | ★ | ★ | ★ | — | 7 |
| **Guo et al.** | ★ | ★ | ★ | ★ | ★ | ★ | — | — | 6 |
| **Banno et al.** | ★ | ★ | ★ | ★ | ★ | ★ | ★ | — | 7 |
| **Kizilkan et al.** | ★ | ★ | ★ | ★ | ★ | ★ | ★ | — | 7 |
| **Porcaro et al.** | ★ | ★ | ★ | ★ | ★ | ★ | ★ | ★ | 8 |
| **Porcaro et al.** | ★ | ★ | ★ | ★ | ★ | ★ | — | — | 6 |
| **Tominaga et al.** | ★ | ★ | ★ | ★ | ★ | ★ | ★ | ★ | 8 |
| **Kim et al.** | ★ | ★ | ★ | ★ | ★ | ★ | — | — | 6 |
| **Misraï et al.** | ★ | ★ | ★ | ★ | ★ | ★ | ★ | — | 7 |
| **Gunda et al.** | ★ | ★ | ★ | ★ | ★ | ★ | ★ | — | 7 |
| **Ohwaki et al.** | ★ | ★ | ★ | ★ | ★ | ★ | ★ | ★ | 8 |
| **Elkoushy et al.** | ★ | ★ | ★ | ★ | ★ | ★ | ★ | — | 7 |
| **Bhojani et al.** | ★ | ★ | ★ | ★ | ★ | ★ | ★ | ★ | 8 |
| **Kim et al.** | ★ | ★ | ★ | ★ | ★ | ★ | ★ | — | 7 |
| **Yoo et al.** | ★ | ★ | ★ | ★ | ★ | ★ | — | — | 6 |
| **Nunez et al.** | ★ | ★ | ★ | ★ | ★★ | ★ | ★ | ★ | 9 |
| **Antunes et al.** | ★ | ★ | ★ | ★ | ★ | ★ | ★ | ★ | 8 |
