# Supplementary material for: Predictive factors of incidental prostate cancer in patients undergoing surgery for presumed benign prostatic hyperplasia: an updated systematic review and meta-analysis
Source: Front Oncol. 2025 Feb 27;15:1561675. doi: 10.3389/fonc.2025.1561675 (PMC11903258; doi:10.3389/fonc.2025.1561675)
Supplement: Supplementary file 2 [file DataSheet1.docx]

**Supplementary Figure**

**Predictive factors of incidental prostate cancer in patients undergoing surgery for presumed benign prostatic hyperplasia: an updated systematic review and meta-analysis**

**Yang Wang, Xiancheng Li, Hua Yang, Chaoshan Yin, Yameng Wu, Xiaoke Chen**

**
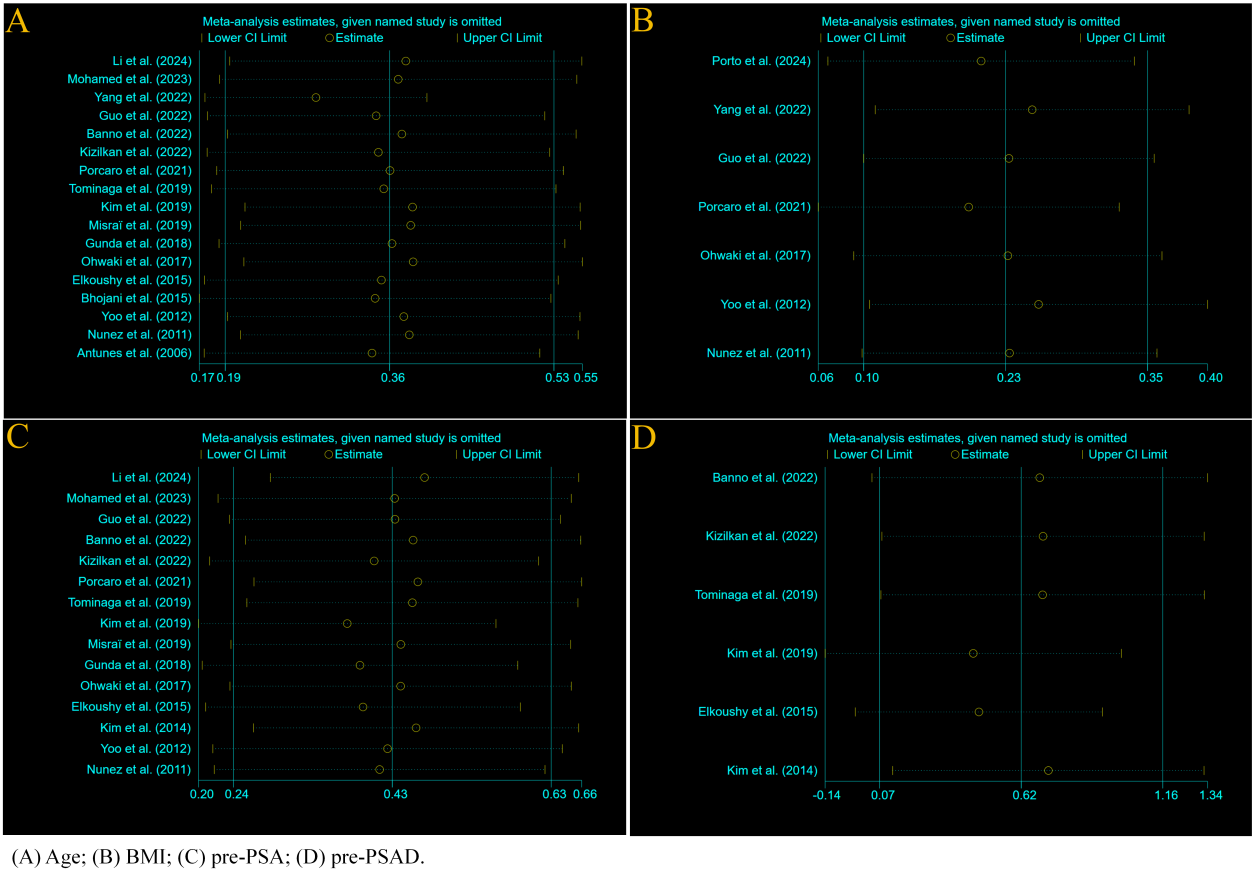
**

**
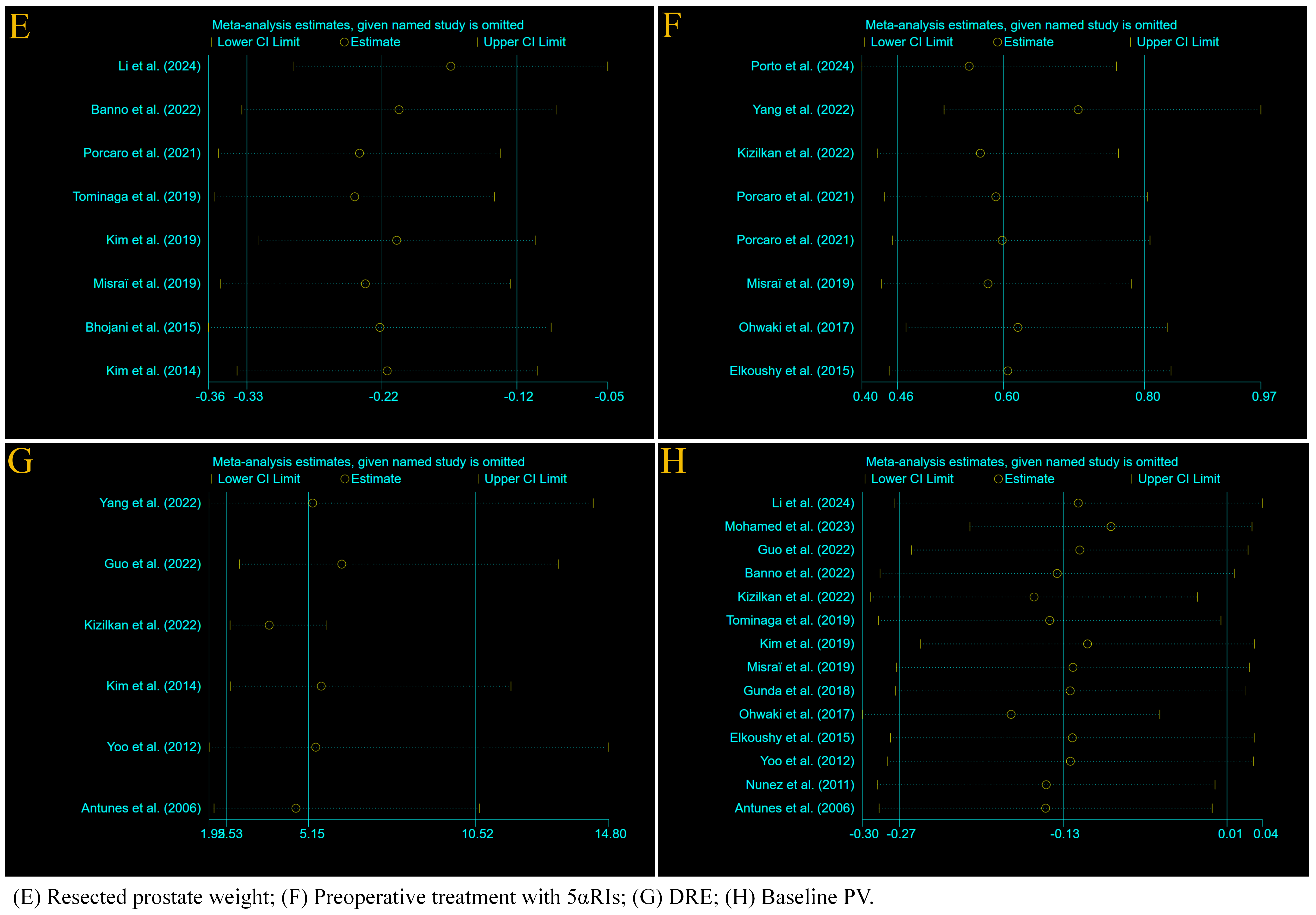

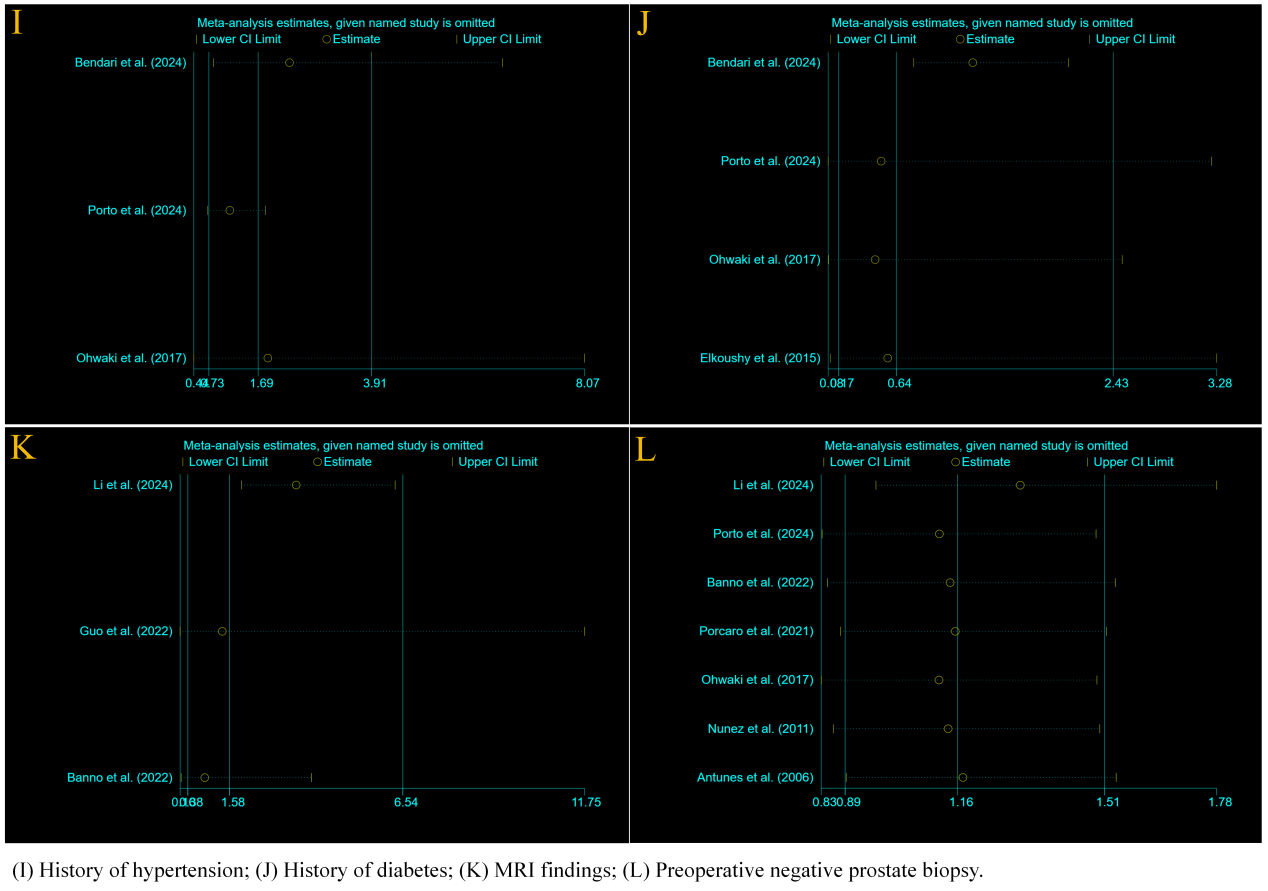
**

**Supplementary Figure 1. Sensitivity analysis of the association between the predictive factors and IPCa risk.** (A) age, (B) BMI, (C) pre-PSA, (D) pre-PSAD, (E) resected prostate weight, (F) preoperative treatment with 5αRIs, (G) DRE findings, (H) baseline PV, (I) history of hypertension, (J) history of diabetes, (K) MRI findings, and (L) preoperative negative prostate biopsy.


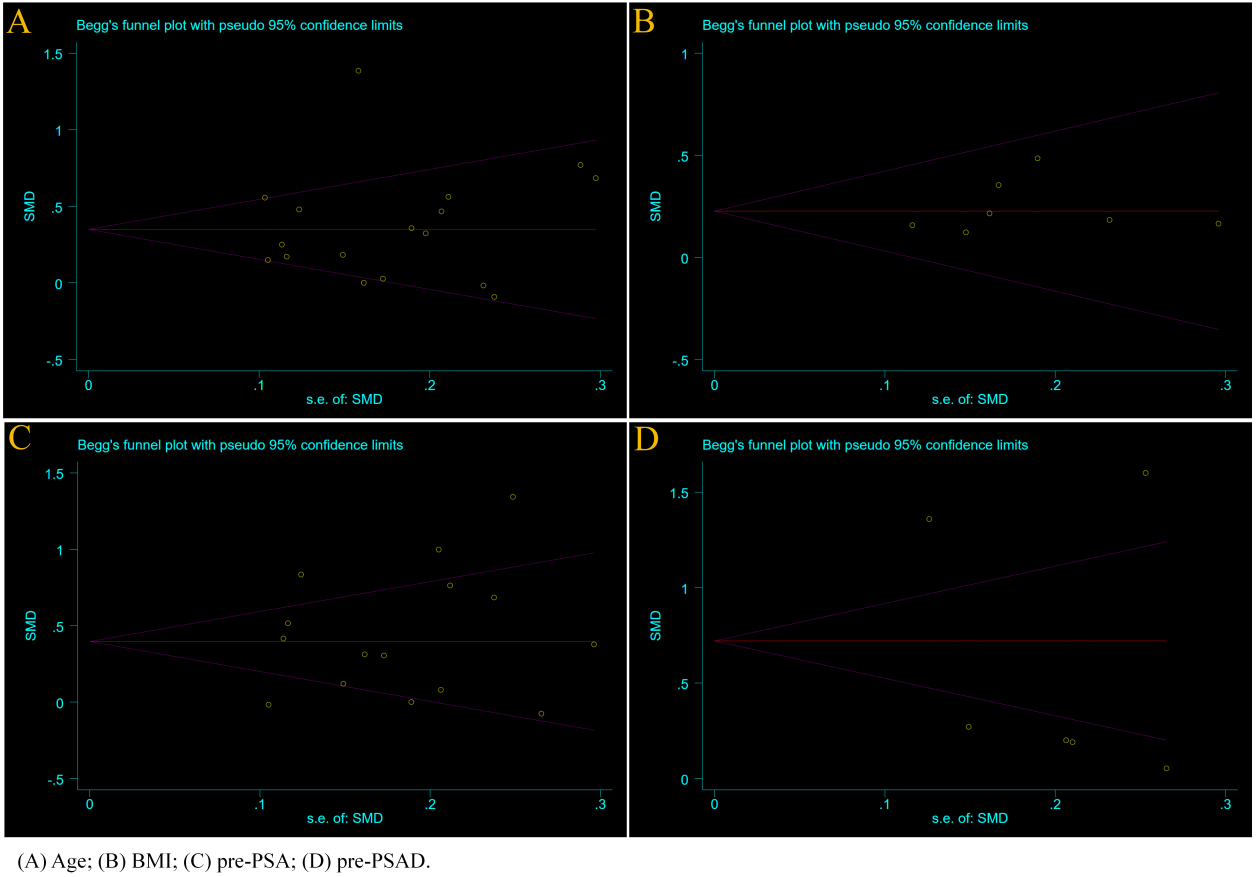


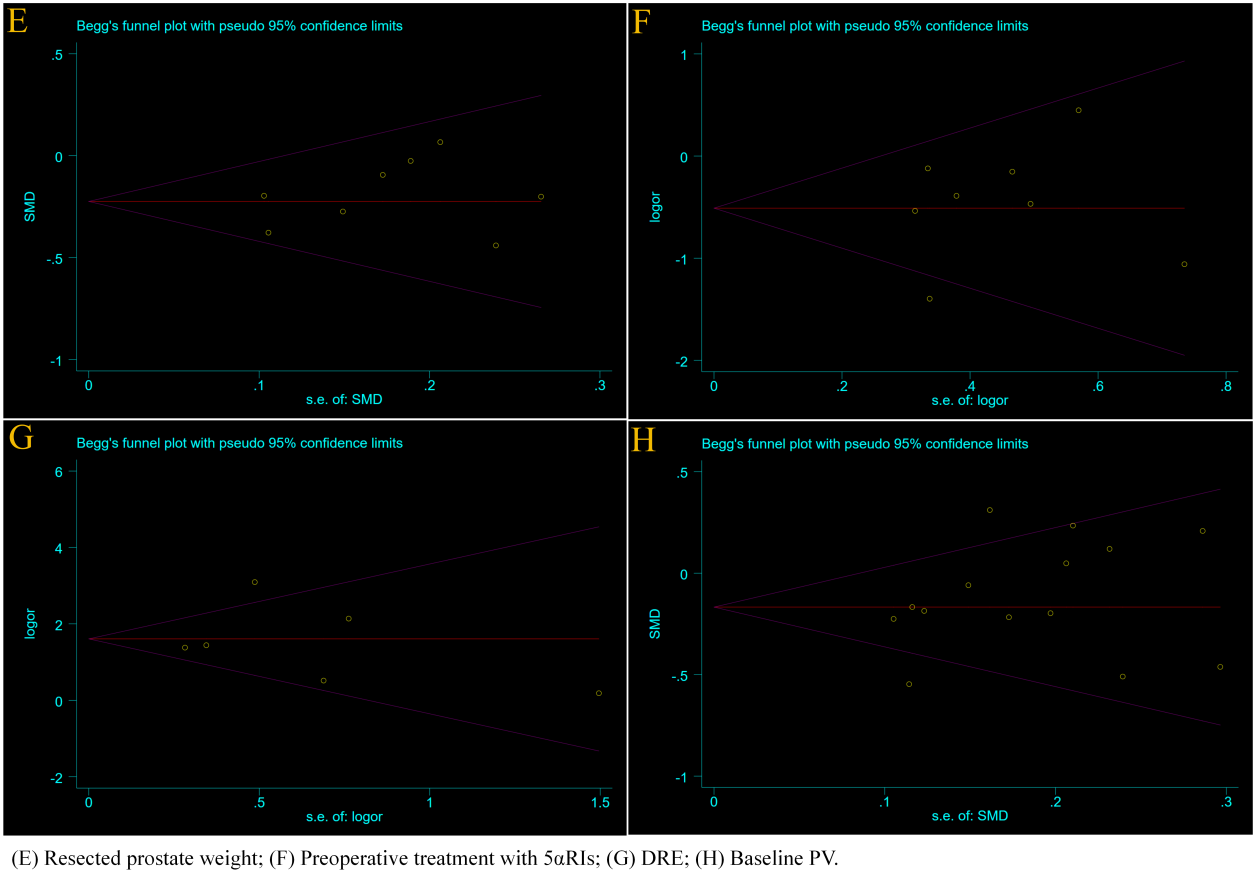


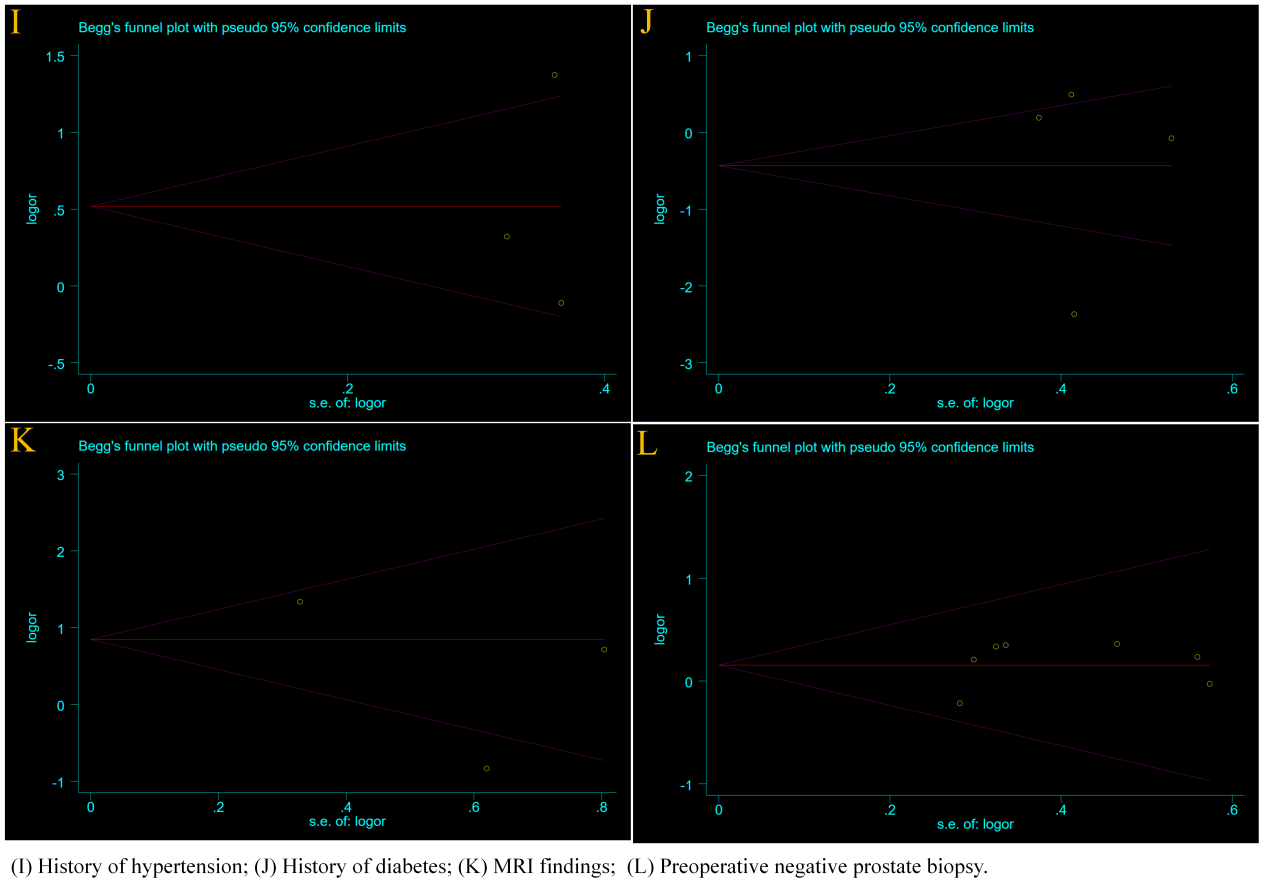


**Supplementary Figure 2. Funnel plot and Begg test for publication bias.** (A) age, (B) BMI, (C) pre-PSA, (D) pre-PSAD, (E) resected prostate weight, (F) preoperative treatment with 5αRIs, (G) DRE findings, (H) baseline PV, (I) history of hypertension, (J) history of diabetes, (K) MRI findings, and (L) preoperative negative prostate biopsy.
